# HBV pgRNA induces chronic inflammation in an IL-1β-dependent manner

**DOI:** 10.3389/fimmu.2026.1812831

**Published:** 2026-06-22

**Authors:** Aikaterini Skeva, Konstantinos Marmanis, Maria Ntinopoulou, Panagiotis Liakopoulos, Eleni Tryfonopoulou, Anna Chalkidou, Stella Arelaki, Katerina Chlichlia, Maria Koffa, Akrivi Chrysanthopoulou, Petros Kolovos, Alexandra Giatromanolaki, Konstantinos Mimidis, Theocharis G. Konstantinidis, Maria Panopoulou

**Affiliations:** 1 Laboratory of Microbiology, Department of Medicine, School of Health Sciences, Democritus University of Thrace, Alexandroupolis, Greece; 2 Laboratory of Cellular and Molecular Biology, Department of Molecular Biology and Genetics, School of Health Sciences, Democritus University of Thrace, Alexandroupolis, Greece; 3 Laboratory of Molecular Immunology, Department of Biological Applications and Technology, School of Health Sciences, University of Ioannina, Ioannina, Greece; 4 Department of Molecular Biology and Genetics, School of Health Sciences, Democritus University of Thrace, Dragana, Alexandroupolis, Greece; 5 Laboratory of Molecular Immunobiology, Department of Molecular Biology and Genetics, School of Health Sciences, Democritus University of Thrace, Alexandroupolis, Greece; 6 2^nd^ Department of Internal Medicine, Democritus University of Thrace, Alexandroupolis, Greece; 7 Laboratory of Pathology, Democritus University of Thrace, Alexandroupolis, Greece; 8 1^st^ Department of Internal Medicine, Democritus University of Thrace, Alexandroupolis, Greece; 9 Laboratory for the Study of Gastrointestinal System and Liver, Democritus University of Thrace, Alexandroupolis, Greece

**Keywords:** chronic hepatitis B, chronic inflammation, HBeAg negative, interleukin-1b (IL-1β), LL-37, pregenomic RNA

## Abstract

**Introduction:**

Chronic hepatitis B (CHB) is a widespread form of hepatitis B infection with advanced complications if unsupervised and untreated. It was previously reported that pregenomic RNA (pgRNA) can be detected in blood circulation as an indirect marker of HBV transcriptional activity. The aim of this study was to investigate the transcriptomic profile of pgRNA-positive patients with CHB in comparison to pgRNA-negative patients and to evaluate its role in innate immunity.

**Materials and methods:**

A total of 88 patients with CHB who were receiving nucleoside analogs (NAs) were enrolled in this study. The viral load and genotype, HBV pgRNA, and biochemical and virological markers were determined. Samples from eight CHB HBeAg-negative patients were sequenced. Four of them were positive for HBV pgRNA, while the rest were negative. These data were processed via bioinformatic tools. Bioinformatic analyses revealed common pathways such as platelet activation and neutrophil degranulation, for which experimental setups were organized. Platelet activation, as well as platelet–neutrophil interactions, was examined. Furthermore, the synthesis and expression of interleukins IL-1β, IL-17A, and LL-37 were studied.

**Results:**

The percentage of patients positive for HBV pgRNA in the study population was 18.1%. With respect to the transcriptomic analysis, which was performed via two distinct analyses, we identified common differentially expressed (DE) genes involved in platelet activation and neutrophil degranulation pathways. Afterwards, the induction of autophagy in platelets and the *de novo* synthesis of the peptide LL-37 were observed in platelets stimulated with HBV pgRNA-positive serum. Furthermore, a combined study of platelet–neutrophil interactions revealed the development of a proinflammatory phenotype characterized by high levels of IL-1β in neutrophils, accompanied by minimal NET formation. In conclusion, those findings were intended to demonstrate the overexpression of IL-1β and LL-37 in the liver tissue of patients with CHB.

**Conclusions:**

This study demonstrated that HBV pgRNA activates platelets in an autophagy-dependent manner. Moreover, the platelet–neutrophil axis is responsible for a proinflammatory phenotype in which IL-1β is overexpressed.

## 1 Introduction

An estimated 250 million people worldwide suffer from chronic hepatitis B (CHB) (1). Chronic hepatitis B is organized into four clinical stages according to EASL, namely: (i) the immune-tolerant phase (IT), (ii) the immune-active phase (IA), (iii) the inactive carrier phase (IC), and (iv) the HBeAg-negative phase (ENEG) (2). The most clinically unstable stages during CHB are the IA and ENEG conditions, given their immunologically active state, which can easily lead to spontaneous illness exacerbation (3). Most of those patients are under treatment and in close surveillance for the assessment of a prominent recession. The mutagenic origin of HBV is closely related to increased incidence of cirrhosis and HCC, which are often present in CHB patients—for example, the 20-year incidence of developing cirrhosis among CHB patients is 30%, and the incidence of developing HCC is 2%–5% per year for cirrhotic patients (4).

Factors implicated in the onset and progression of liver fibrosis lead to a pathological imbalance between HBV genomic persistence and the activation of local immune responses. The crosstalk of liver parenchymal cells (hepatocytes) and nonparenchymal cells, such as Kupffer cells (KCs) and liver sinusoidal endothelial cells (LSECs), increases the permeability and infiltration of innate immune cells (neutrophils and monocytes) and T cells and ultimately triggers mechanotransduction of hepatic stellate cells (HSCs). Various immonological conditions are involved in adaptive and innate immunity, which facilitate chronic inflammation in the liver microenvironment. The synergistic collaboration of innate immunity with adaptive immunity appears to play an important role in sustaining chronic inflammation. HbsAb can bind to HbsAg and control the spread and phagocytosis of HBV-infected cells (5). Dendritic cells (DCs) express and recognize the infected cells through Toll-like receptors (TLRs) and produce type I IFN and other proinflammatory cytokines. However, after 24 h of exposure to the virus, the DCs are exhausted and downregulated because of the ability of HBV to cover itself with host proteins and escape innate immunity. Persistent chronic inflammation is induced by the polarization of macrophages to the M2 anti-inflammatory phenotype and the secretion of the cytokines IL-6, IL-1, and TNF-a by KCs. Furthermore, KCs enhance the development of Tregs and inactivate both follicular helper T (fhT) cells and B cells, leading to the inhibition of HBsAb formation. On the other hand, activated neutrophils form extracellular traps (NETs) by producing a variety of bioactive peptides and proteins. IL-1β-bearing NETs contribute to local infiltration. By expressing and secreting IL-12, NETs stimulate NK cells to produce IFNγ and promote TH1 differentiation, whereas through IL-17a or LL-37 they activate HSCs and contribute to CHB-related liver injury. Finally, neutrophils interact with fibroblasts, leading them to acquire a profibrotic phenotype (6).

One of the most implicated cells in the preservation of chronic liver inflammation is the longitudinal existence of CD8+ T cells. HBV-differentiated T cells are part of adaptive cellular immunity; their activation during the acute phase of HBV brings them in front of various antigens expressed from the complicated HBV genome (HBsAg, small, medium, large, HBeAg, Hbx, and HBcrAg) and sets them aside from the effective recognition and elimination of viral particles. In addition, the persistence of cccDNA in the liver microenvironment continues to reinforce viral transcriptomic activity and excludes CHB patients from viral clearance.

During durable viral replication activity and the eminent immunological factors that coexist in the liver microenvironment, platelets seem to have distinguished implications in liver inflammation. Through immunothrombosis, they interact with activated CD8+ T cells and attract them to the liver microenvironment to recognize and destroy HBsAg-expressing hepatocytes (7). In parallel, platelets, as regulatory cells that release diverse molecules acting as inflammatory attractants and accumulating in the liver microenvironment, and various immune cells, such as neutrophils and macrophages, can deteriorate the liver’s status from necroinflammation to fibrosis (8, 9).

Furthermore, platelets release serotonin, which regulates sinusoidal microcirculation by reducing local blood flow and thereby contributes to worsening liver damage (10, 11). Experimental cohorts of chronic HBV patients have been previously used to compare the regulatory role of antiplatelets and anticoagulants in the development of fibrosis and HCC. Interestingly, Lee et al. reported that the incidence of HCC was lower in patients treated with antiplatelets (hazard ratio, 0.34; *p* = 0.01) (12). Mechanistically, this is attributed to the combination of aspirin, which blocks thromboxane A2 production, and clopidogrel, which blocks the P2Y12 ADP receptor, leading to reduced accumulation of virus-specific CD81+ T cells in the liver and ameliorating inflammation (13).

In addition, platelets, through specific TLRs on their surface, are able to interact with pathogens (14). They recognize the pathogen and secrete a granule containing antimicrobial peptides such as the LL-37 molecule, which acts against infected cells (15). In addition, platelets can secrete ROS against pathogens and restrict their spread through sinusoidal thrombus formation (16). Platelets either acquire their cargo from megakaryocytes or synthesize it using existing mRNAs and a functional spliceosome (17). The platelets’ exposure to an inflammatory microenvironment can increase the expression of surface integrins and stimulate their role in inflammation—for example, platelets’ stimulation with LL-37 has been found to increase the expression of surface molecules implicated to pathogen recognition and thrombosis formation. Such molecules are the TLR-2 and TLR-4 receptors and the CD41 (integrin aIIb) and aIIβ3 integrin in their active conformation (18). To date, there has been no experimental evidence confirming either the release of LL-37 from platelets in the infected liver microenvironment or its action against infected hepatocytes.

The continuous activation of platelets during thrombosis and hemostasis is closely related to the mechanism of autophagy. Autophagy is active in resting platelets and is overrepresented when platelets participate in the immunothrombosis pathway. Studies have shown that knockout of Atg7, a primary protein of the autophagosome with high significance for LC3-B expression, led to reduced aggregation and granule cargo packaging in *ex vivo* experimental cohorts (19). However, platelets exhibit a disorganized form of cellular behavior. Although they cannot synthesize proteins, they can interact with cells of the innate immune system and promote either proinflammatory or profibrotic phenotypes. Accordingly, we examined the proinflammatory and fibrotic profiles of activated platelets enriched with either HBV pgRNA-enriched serum or the absence of HBV pgRNA in the serum and its impact on neutrophil stimulation. We sought also the importance of staining a CHB liver tissue for the presence of either LL-37 and IL-1β in the liver microenvironment.

We deployed methodologies such as RNA sequencing and *in vitro* cohorts to investigate the interactions of innate immunity in chronic hepatitis through the neutrophil–platelet axis. The presence of HBV pgRNA was a significant parameter to study among our samples, and its contribution to innate immunity has emerged, providing new insights into our research.

## 2 Materials and methods

### 2.1 Patients and biological samples

In this study, we examined 88 plasma samples collected between January 2022 and August 2023 in the hepatology outpatient clinic of the First Department of Internal Medicine. Each sample aligns to a patient with CHB hepatitis who has been receiving NA treatment for at least 1 year. Patients with HCC as well as patients with concomitant infections caused by hepatitis D virus, hepatitis C virus, or human immunodeficiency virus were excluded. All patients were Caucasian and examined for the HBV genotype, which was predominantly positive for the D type. All of the plasma samples were stored in aliquots at -80 °C and thawed once for analysis. All of our patients were under NA treatment and negative for HBeAg, with some showing a detectable HBV DNA load either due to early therapy initiation or due to no response to NA therapy. There were 22 HBV-DNA-load-positive individuals.

### 2.2 Real-time PCR for HBV genotype determination

All samples were tested for HBV genotypes based on qualitative determination by real-time PCR. Considering the distinct geographical dominance of HBV genotype D in the Mediterranean region, we tested all of our samples via an HBV genotype A–D real-time kit (Sacace, Como, Italy).

### 2.3 Real-time PCR for HBV RNA quantification

For HBV RNA quantification, we used the quantitative method of Laras et al. (20) with three predefined pairs of primers for the detection of preC mRNA, CP-directed transcript (preC mRNA plus pgRNA), and DNA contamination.

RNA was extracted from 400 µL of plasma using the NucleoSpin Virus DNA/RNA Kit (Macherey Nagel GmbH and Co., Düren Germany), and 10 µL of eluate was pretreated for possible gDNA contamination and reverse-transcribed using the PrimeScript RT Reagent Kit with gDNA Eraser (Takara Bio, CA 91043, USA). We used 5 µL of eluate for the reverse transcription reaction and cDNA synthesis via the antisense BC1 primer (5’-GGAAAGAAGTCAGAA GGCAA, nt1974-1955). One microliter from the cDNA reaction was subsequently used for each of the three different RT-PCRs, with BC1 as the common 3’ primer, PCP (5′-GGTCTGCGCACCAGCACC, nt1796--1813) as the 5’ primer for preC mRNA detection, PGP (5′-CACCTCTGCCTAATCATC, nt1826--1843) as the 5’ primer for CP-directed transcription, and M3 (5′-CTGGGAGGAGTTGGGGGAGGAGATT, nt1730--1754) as the 5’ primer for DNA contamination. The reaction mixture was composed of 0.32 mM MgCl, forward/reverse primer (0.5 mM), probe FL (4 mM), probe LC (8 mM) (**Supplementary Table 1**), H_2_O, and Taq Polymerase/dNTP solution from the LightCycler^®^ FastStart DNA Master HybProbe (Roche Diagnostics GmbH, Mannheim). The pgRNA levels were calculated by subtracting preC mRNA from the total CP-directed transcript for each sample, and DNA contamination was detected by examining the samples for CP-directed transcription and/or cDNA products.

To standardize the precision of the assay, we used sequentially diluted samples of a customized cloned plasmid containing the desired nucleotide sequence. After standardizing the assay, we determined the lower limit of detection (LLD) at 262 (2.42 log10) copies/mL (c/mL) and the LQD at 322 (2.51 log10) copies/mL (c/mL) serum.

### 2.4 Cell-free RNA sequencing

We collected plasma samples from all of the HBV-pgRNA-treated patients and performed RNA extraction according to the TRIzol (Invitrogen, Waltham, MA, USA) protocol. We further proceeded with RNA sequencing by using the TruSeq RNA Library Prep Kit v2 (Illumina Inc., San Diego, CA, USA). We loaded 1 µg of cDNA for each sample and ran it on the Illumina MiSeq platform. For this procedure, we collected samples from four patients belonging to the following categories: (I) HBV pgRNA positive, (II) HBV pgRNA negative, and (III) healthy individuals.

### 2.5 Retrieval of data from GEO

We downloaded data associated with HBV-related HCC malignancy from the GEO database with the accession number GSE57381 and compared HBV pgRNA positivity with possible indices related to the development of HCC.

### 2.6 Raw data editing

We further proceeded with fastq data editing, which started by trimming the adapters of the forward and reverse reads. For this process, we used the bioinformatic tool TrimGalore (21). In addition, we assessed sequence quality after trimming via the fastp tool (22). Afterwards, we aligned our reads on the last edition of the human genome GRCh38/hg38 by using HISAT2 (23). Finally, we used the HTseq count bioinformatics tool to determine the genomic locations of our reads in the human genome (24).

### 2.7 Differentially expressed genes and count editing

First, the counts were analyzed through PCA to reveal possible intragroup interactions between the four groups, namely: (I) HBV pgRNA positive, (II) HBV pgRNA negative, (III) healthy individuals, and (IV) HBV-related HCC patients. The differentially expressed (DE) genes were identified on the basis of the parameters |logFC| ≥1 and *p <*0.05 after the DESeq2 R package was applied.

The common genes found between the four categories were plotted on a Venn diagram, and the pheatmap and ggplot2 packages were used to depict intragroup mean value differences (25–28).

### 2.8 Enhanced pathways of the DE genes

The common DE genes were excessively analyzed and introduced as leading contributors to the pathways characterized as being enhanced after gene set expression analysis (GSEA). GSEA was performed using the clusterProfiler, R package (29).

### 2.9 Platelet stimulation with CHB patient serum

We further stimulated the platelets collected from healthy individuals with HBV pgRNA-positive or HBV pgRNA-negative serum (30). We cocultured 200 µL of platelets diluted in HEPES (pH 8) with 1,000 µL of DMEM and 5% serum from the four different conditions in six-well plates, namely: (I) healthy individuals, (II) those treated with clarithromycin (positive control), (III) those positive for HBV pgRNA, and (IV) those negative for HBV pgRNA. We incubated the samples at 37 °C in 5% CO_2_ and 18%–21% O_2_ for 90 min. Finally, we collected the supernatant and the stimulated platelets and used them for RNA extraction or antimicrobial and migration assays.

### 2.10 Real-time PCR of autophagy markers and the LL-37 peptide in platelets

For autophagy detection, we created three pairs of primers to detect each of the three most informative genes involved in the autophagy pathway. The first gene is an autophagosome initiation protein named BECLIN-1 (forward primer: 5’ GGACACTCAGCTCAACGTCA 3’, reverse primer: 5’ AGCCTGGACCTTCTCGAGAT 3’), the second gene is LC3-B (forward primer: 5’ GGTGAGAAGCAGCTTCCTGT 3’, reverse primer: 5’ TCTCCTGGGAGGCATAGACC 3’), and finally the third gene is P62 (forward primer: 5’ TGTGTAGCGTCTGCGAGGGAAA 3’, reverse primer: 5’ AGTGTCCGTGTTTCACCTTCCG 3’). The following steps highlight the preparation of the three distinct master mixes in a final volume of 20 µL. Each master mixture contained 8.2 µL H_2_O, 10 µL SYBR Green (KAPA SYBR^®^ FAST qPCR Master Mix (2X), Cape Town, South Africa), 0.4 µL forward primer (0.5 mM), and 0.4 µL reverse primer (0.5 mM). The thermal profiles of the abovementioned genes are depicted in **Supplementary Table 1**. Finally, for the LL-37 quantification, we used the following pair of primers: forward primer: 5’ GGGAAGCTTGTCATCAATGG 3’, reverse primer: 5’ CATCGCCCCACTTGATTTTG 3’, whereas as a housekeeping gene, we demonstrated the expression pattern of the GAPDH gene (forward primer: 5’ GGGAAGCTTGTCATCAATGG 3’, reverse primer: 5’ CATCGCCCCACTTGATTTTG 3’). The thermal profile is depicted in **Supplementary Table 2**. The primers used were designed using the NCBI Primer Blast tool.

### 2.11 Proinflammatory profile of NETs after stimulation with HBV pgRNA +/- activated platelets

We studied the proinflammatory pathway between neutrophils and those already activated from CHB serum PLTs. In six-well plates, we incubated 200 µL of healthy isolated neutrophils with 1,000 µL of DMEM and stimulated them with one of the following four conditions: (I) the PLT supernatant was first activated with healthy individual serum, (II) the PLT supernatant was first activated with asthma serum, (III) the PLT supernatant was first activated with HBV pgRNA-positive serum, and (IV) the PLT supernatant was first activated with HBV pgRNA-negative serum. Asthma serum was used as a positive control to indicate the proinflammatory profile. We incubated the samples at 37 °C in 5% CO_2_ and 18%–21% O_2_ for 3 h. The NET structures were further used for downstream assays, such as RNA isolation and IL-1β/IL-17A qRT–PCR.

### 2.12 Immunofluorescence

For the immunofluorescence assay, we cultivated 100 µL of stimulated platelets diluted in HEPES with 400 µL of DMEM in 24-well plates containing polyA-coated glass. We performed four assays with pretreated platelets, including those with 5% serum, namely: (i) healthy individuals, (ii) clarithromycin as a positive control, (iii) patients with HBV pgRNA-positive serum, and (iv) patients with HBV pgRNA-negative serum. We further incubated the pretreated platelets for 90 min at 37 °C, 5% CO_2_, and 18%–21% O_2_ and fixed them on the surface of the polyA-coated glass with 500 µL of 4% formaldehyde by incubating them for 15 min. Consequently, we washed the samples with 150 µL of 1× PBS and incubated them for 5 min. We repeated the process twice, and after the third incubation, we transferred the polyA-coated glasses to 150 µL of 0.5% Triton X and incubated them sharply for 1 min to increase the permeability of the cells. We continued with triple incubation at room temperature for 5 min with 150 µL of 1× PBS. After the last incubation, 5% goat serum was added, and the samples were blocked for 30 min at room temperature. A total of 1 h of incubation with 20 µL of CD41a (CD41-APC, P2, mouse antihuman, Beckman Coulter, Indianapolis, IN, USA) and 1 h of incubation with 1/300 primary antibody against LC3-B (rabbit polyclonal antihuman, Origene Technologies, Rockville, MD, USA) were performed. We continued with three repeats of 1× PBS washes for 5 min and ended the procedure with 1 h of incubation with 1/400 of the secondary antibody (goat anti-rabbit CF647, Biotum, San Francisco, CA, USA). We subsequently incubated a second batch of stimulated platelets under the same conditions with the primary LL-37 antibody (mouse monoclonal anti-human, Santa Cruz Biotechnology, Dallas, TX, USA), incubated them for 1 h at room temperature, and then incubated them for 1 h at room temperature with the secondary (rabbit anti-mouse CF488A, Biotum, San Francisco, CA, USA) antibody specific for LL-37. We finalized the procedure with three repeats of 1× PBS washes, lasting for 5 min each, and finished by mountaining the polyA-coated glasses on top of the microscopy slides and performing immunofluorescence.

We continued with immunofluorescence analysis of the proinflammatory profile depicted from the communication between NETs and the PLT supernatant. For the immunofluorescence assay, we cultivated 100 µL of healthy isolated neutrophils in 400 µL of DMEM in 24-well plates containing poly-A-coated glass. We performed four assays with pretreated neutrophils, including those with 5% PLT supernatant, namely: (I) healthy individual serum, (II) asthma serum, (III) HBV pgRNA-positive serum, and (IV) HBV pgRNA-negative serum. We next used the proinflammatory marker IL-1β (rabbit, Origene Technologies GmbH, Herford, Germany) and the neutrophil elastase antibody (mouse, Origene Technologies GmbH, Herford, Germany) for the assessment of NET structure formation and DAPI (Biotium, Fremont, CA, USA) as a DNA detection index.

We proceeded to further experiments inhibiting the effect of HBV pgRNA in the healthy platelets and incubated 10 μL of HBV pgRNA-positive serum with 2 μL RNase (Thermo Fisher Scientific Inc.) at 37 °C, 5% CO_2_, and 18%–21% O_2_ for 30 min. We continued with the steps described in Section 2.9. In addition, we restricted the action of the free IL-1β in the serum to test if the IL-1β signal does not interfere with the platelets’ activation. We incubated 20 μL of HBV pgRNA-positive serum with interleukin 1β receptor (IL-1R) antagonist, anakinra, in a final concentration of 0.1 mg/mL and incubated it for 30 min at RT before we proceeded to the steps described in Section 2.9.

### 2.13 Migration assay of fibroblasts

The fibroblasts were cultured in a 24-well cell culture plate (SPL Life Sciences, Kyonggi-do, Republic of Korea) until 90% confluency was reached. A wound was subsequently created with a scratcher tip (SPL Life Sciences, Kyonggi-do, Republic of Korea), and stimulation of the fibroblasts (C-12360, PromoCell, Heidelberg, Germany) was performed. Different stimulation methods, such as fibroblasts treated with (I) healthy individuals’ serum, (II) clarithromycin (positive control), (III) HBV pgRNA-positive serum, or (IV) HBV pgRNA-negative serum, have been used. After 20 h of incubation, the migration capacity of the fibroblasts was assessed using May–Grunwald Giemsa staining.

### 2.14 May–Grunwald Giemsa staining

The fibroblasts were stained with May–Grunwald for 5 min at RT and washed with water to further stain them with Giemsa stain (dilution 1:10) for 20 min at RT. Afterwards, a final wash with water was performed, and the samples were dried naturally. The cells were inspected using an OLYMPUS upright microscope (Olympus Corporation, Tokyo, Japan) equipped with a 4x air lens camera (0.10 NA) (PLCN4X/0.1, Olympus Corporation, Tokyo, Japan). For further image modification, OLYMPUS cellSens Entry software (Olympus Corporation, Tokyo, Japan) was used in cooperation with Fiji software version 2.9.0 (31).

### 2.15 Antimicrobial assay

We studied the antimicrobial activity of the stimulated pletelets by coculturing their supernatant with seven serial dilutions of *Escherichia coli* (OD 2) in liquid broth culture medium. In addition, we placed 50 µL of each dilution with 50 µL of previously stimulated platelet supernatants for each of the following conditions: (I) healthy serum, (II) clarithromycin, (III) HBV pgRNA-positive serum, and (IV) HBV pgRNA-negative serum. The mixture was subsequently incubated for 24 h at 37 °C, 5% CO_2_, and 18%–21% O_2_.

Afterwards, we cultured the 6th *E. coli* dilution in solid MacConkey agar medium, and a 24-h incubation was performed. The Petri dishes were evaluated for *E. coli* colony development.

### 2.16 IL-1β and IL-17A real-time PCR

To study the relative expression of IL-1β, we isolated RNA from NETs created after stimulation with the PLT supernatant, prepared cDNA (PrimerScript, Takara Bio Inc., Shiga, Japan), and performed qRT-PCR for IL-1β detection. The primer pair was designed for the forward and reverse primers as follows: forward primer: 5’ GCTCTGGGATTCTCTTCAGC 3’; reverse primer: 5’ TCATTGCCACTGTAATAAGCCA 3’. The signal was detected using SYBR Green dye (Kapa SYBR Fast, Kapa Biosystems, Cape Town, South Africa). We standardized the thermal profile on the basis of the primers’ Tm values (**Supplementary Table 3**). Similarly, we prepared a qRT-PCR mixture for the detection of IL-17a using the following primers: forward primer: 5’ TGGTGTCACTGCTACTG 3’ and reverse primer: 5’ CATTGCGGTGGAGATTC 3’. Finally, we used a Kapa SYBR fast kit to detect the IL-17A signal (Kapa Biosystems, Cape Town, South Africa) by following the thermal profile presented in **Supplementary Table 4**.

### 2.17 Immunohistochemical staining

Serial 3-μm sections were cut from formalin-fixed, paraffin-embedded (FFPE) tissue blocks using a semi-automated microtome (Myr M-240, Tarragona, Spain). Human liver tissue sections obtained from patients with chronic hepatitis B (CHB) were incubated at 80 °C for 30 min and subjected to heat-induced antigen retrieval by microwave treatment for 15 min in EnVision FLEX Target Retrieval Solution, High pH, pH 9 (DM828, DAKO/Agilent, Glostrup, Denmark). Endogenous peroxidase activity was blocked by incubation with EnVision FLEX Peroxidase-Blocking Reagent for 10 min.

All tissue samples were stained using the peroxidase-based EnVision FLEX Mouse/Rabbit Detection System, High pH (DAKO/Agilent, Glostrup, Denmark). Sections were incubated overnight with a mouse monoclonal anti-human LL-37 primary antibody (Santa Cruz Biotechnology, Dallas, TX, USA) at a dilution of 1:50 or with a rabbit anti-IL-1β primary antibody (Origene Technologies GmbH, Herford, Germany) at a dilution of 1:100. Subsequently, the sections were incubated with EnVision FLEX/HRP polymer reagent (SM802, DAKO/Agilent, Glostrup, Denmark) for 30 min.

Visualization of the antigen–antibody complex was performed using EnVision FLEX diaminobenzidine (DAB) chromogen (DM827, DAKO/Agilent, Glostrup, Denmark). Sections were counterstained with EnVision FLEX hematoxylin for 5 min, mounted, and examined under a light microscope. Immunohistochemical images were captured using a Nikon Eclipse 50i light microscope (Nikon Instruments Inc., Tokyo, Japan) equipped with a Basler digital camera (Basler AG, Ahrensburg, Germany). Image acquisition was performed using pylon Viewer software.

### 2.18 Statistical analysis

The median values between the variables were assessed through the Kruskal–Wallis (ANOVA) multiple comparisons test for their statistical significance. The threshold for the statistically accepted values was set at 0.05, and the analysis was performed using the R packages stats, limma, and GraphPad Prism software (32, 33).

## 3 Results

### 3.1 Patient status

All of the CHB patients tested for the HBV genotype were positive for the delta type and negative for HBeAg. HBV pgRNA was detected in 16 out of the 88 samples (18.1%), and the median HBV load was 776.25 c/mL (2.89 log10).

**Figure 1:**
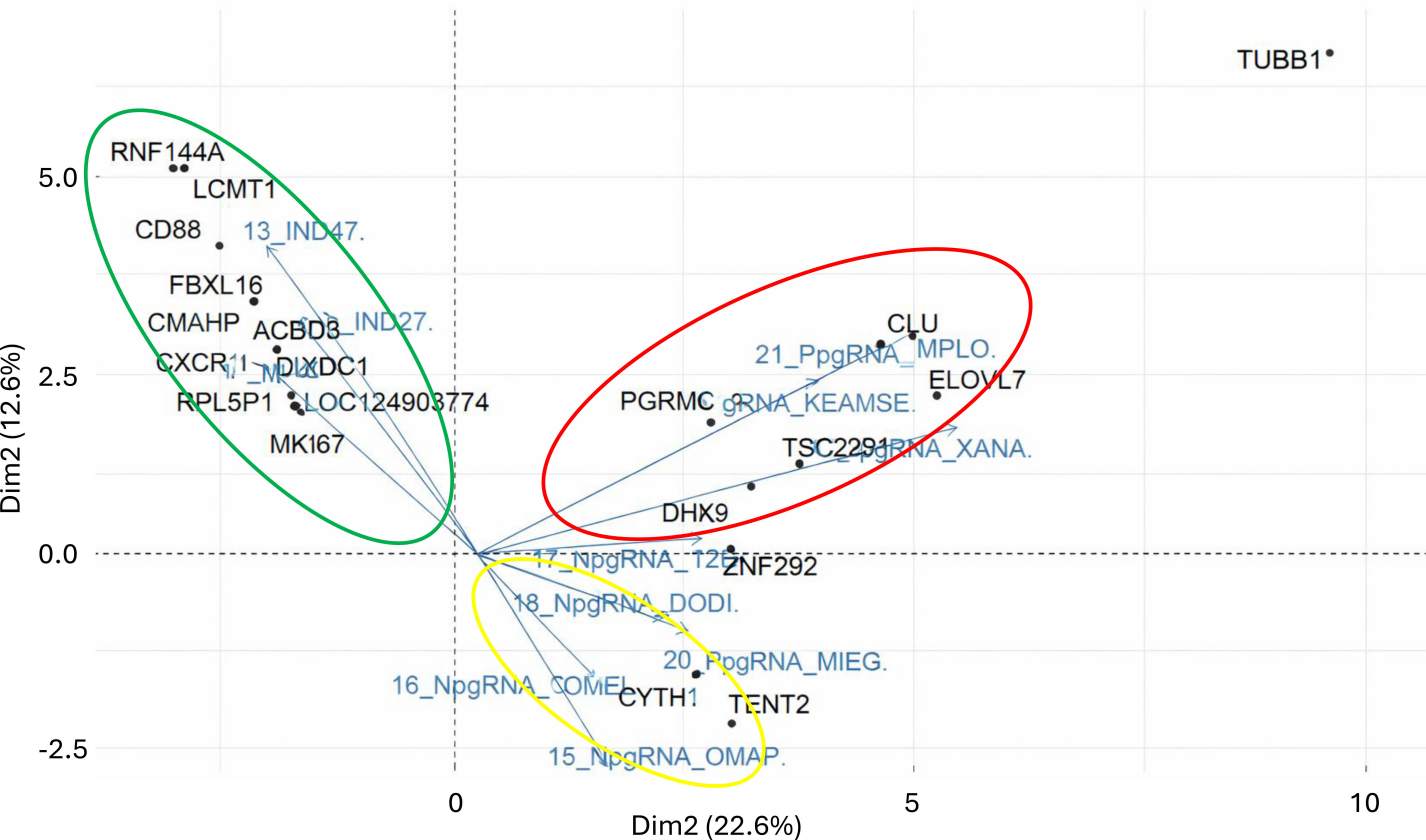
PCA of the studied groups depicting transcriptomic profile differences between the CHB patients who were positive for the HBV pgRNA marker and those who were not compared with healthy individuals.

**Figure 2:**
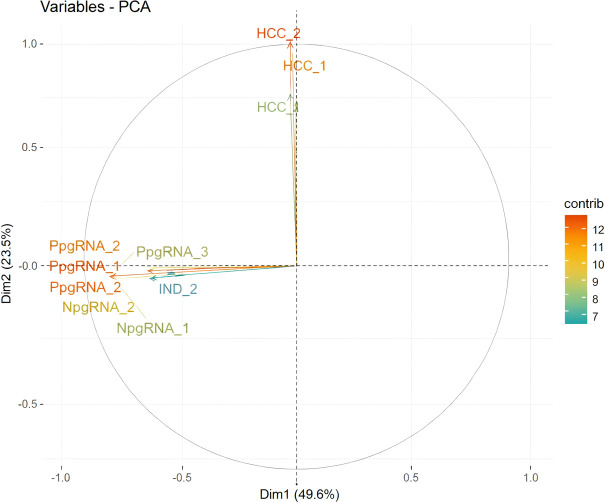
PCA of the CHB patients considered and treated as a common group compared with the HBV-related HCC patients retrieved from the GEO study (GSE75381). The PCA revealed no differences between the CHB patients and the healthy individuals despite their distinct differences from the HBV-related HCC patients.

The patients were separated into two categories according to their HBV pgRNA serum persistence. The two CHB categories were first compared with each other and a healthy study group. The results of the PCA revealed distinct differences between the HBV pgRNA-positive and HBV pgRNA-negative individuals (**Figure 1**). However, when we analyzed the HBV-related HCC samples, the presence of HBV pgRNA was not different between the HBV pgRNA-positive and HBV pgRNA-negative groups. Therefore, we continued the analysis by considering the HBV pgRNA-positive and HBV pgRNA-negative patients as the CHB category and compared them with the healthy individuals and the HBV-related HCC patients (**Figure 2**).

We continued with the second analysis, as there was illness development between the two groups; however, the pathways identified from the first analysis were also informative for our decision to select the appropriate paths to study, and they are reported in **Supplementary Figure 1**.

### 3.2 Transcriptomic analysis

**Figure 3:**
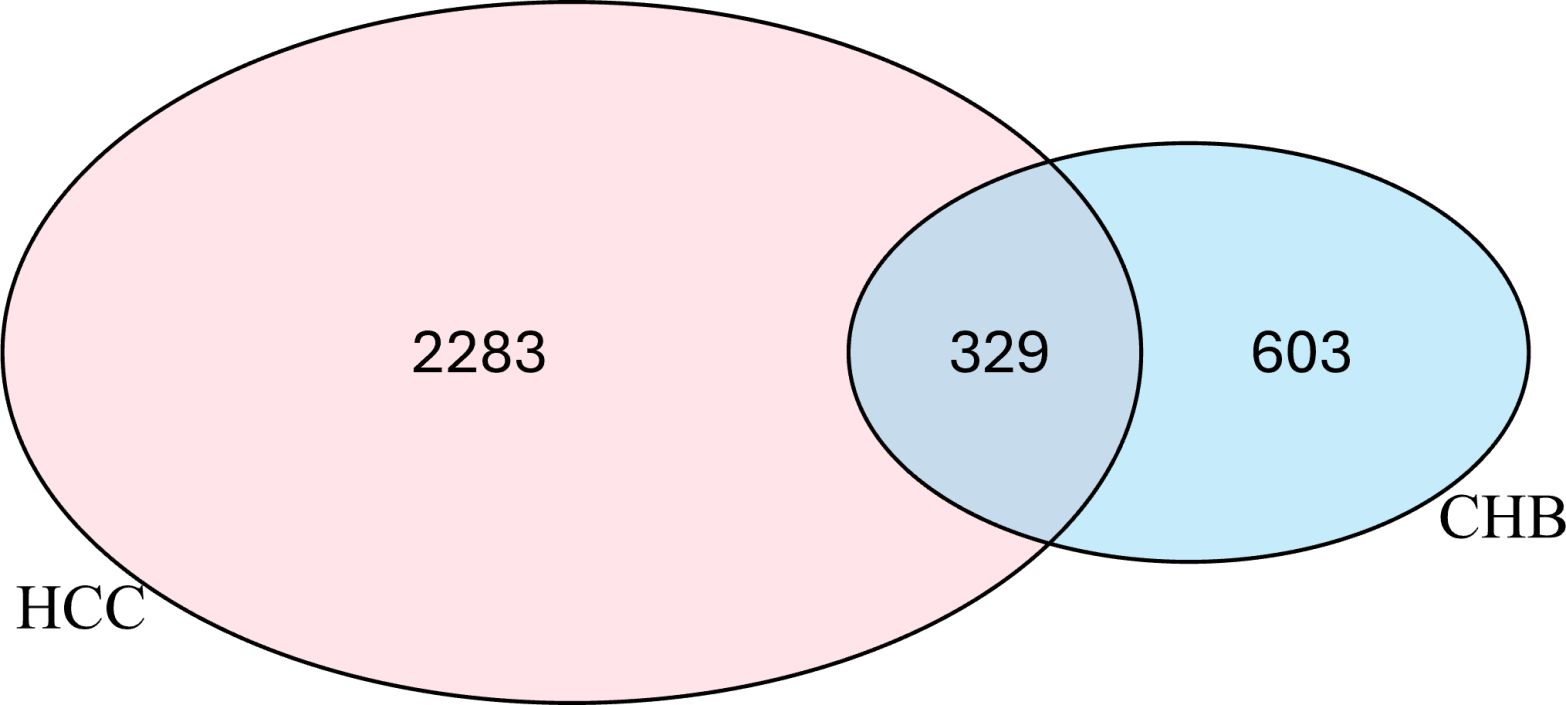
Venn diagram comparing the differentially expressed genes found in the CHB- and HBV-related HCC groups by depicting the common genes between those groups.

**Figure 4:**
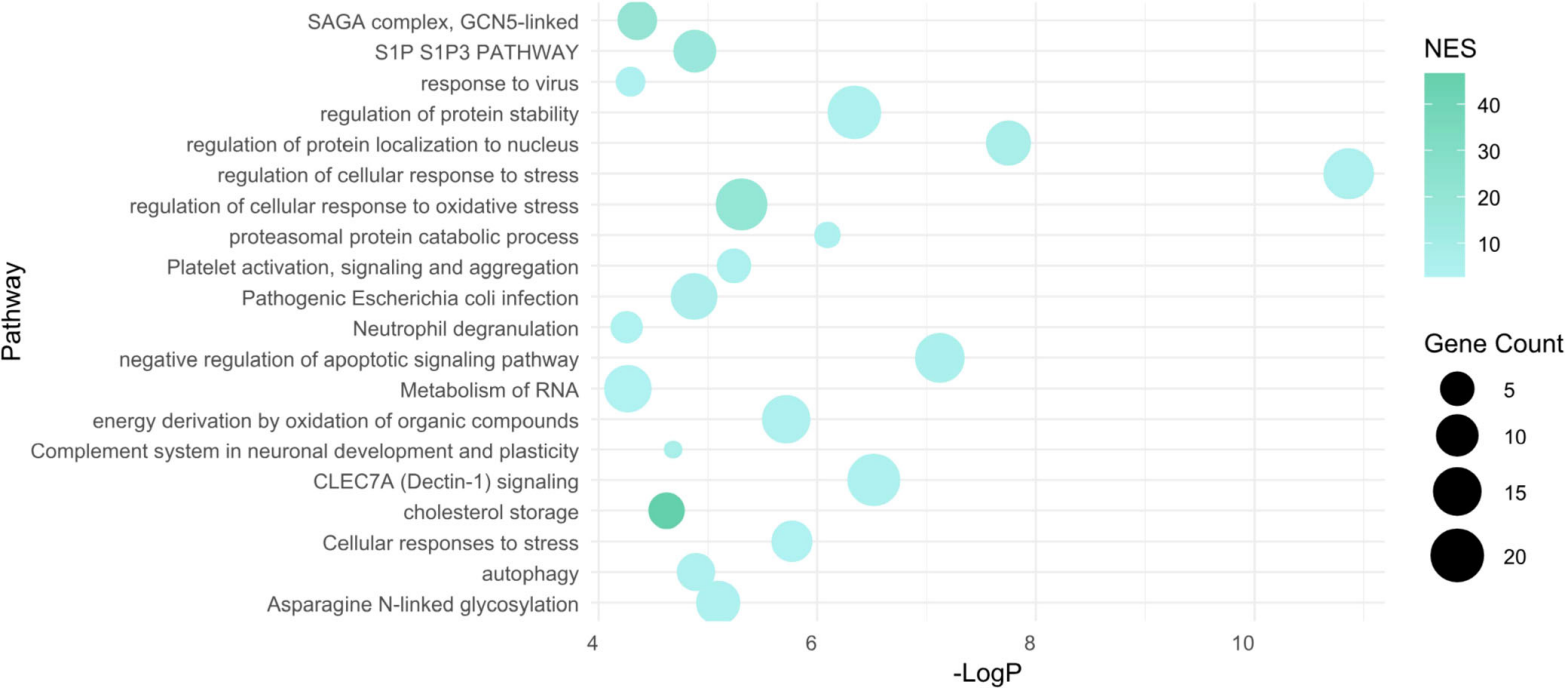
Bubble plot of enhanced pathways associated with the differentially expressed genes.

The results from the transcriptomic analysis revealed 329 genes common to both the CHB patients and the HBV-related HCC patients (**Figure 3**, Venn diagram). In both the CHB patients and the HBV-related HCC patients, the DE genes were upregulated, and the enriched pathways, which were statistically significant at *p <*0.001, were further analyzed for their contribution to the two groups. In total, 20 pathways were identified via GSEA (bubble plot), the majority of which are related to cellular sustainability and cellular recycling (**Figure 4**). Pathways such as (I) the proteasomal protein catabolic process, (II) the negative regulation of the apoptotic signaling pathway, (III) the regulation of cellular responses to stress, (IV) the regulation of protein stability, (V) RNA metabolism, and (VI) autophagy are linked to the regulation of cellular catabolism and to cellular sustainability.

Furthermore, these pathways, which are associated with illness and longevity, are differentially expressed and contribute to the involvement of various immune cells and infectious factors in liver inflammation. The following pathways—(I) platelet activation and aggregation, (II) regulation of the cellular response to oxidative stress, and (III) neutrophil degranulation—contribute to illness chronicity. From the abovementioned pathways, we focused on the platelet activation and aggregation pathway, which was also found enriched between the CHB HBVpgRNA +/- patients’ GSSEA analysis (**Supplementary Figure 1**). In addition, we further studied and analyzed the abovementioned pathways by *in vitro* procedures to unveil possible interactions between neutrophils and platelets during chronic inflammation in the liver microenvironment.

### 3.3 Platelet functions in chronic inflammation

After the stimulation of isolated platelets from healthy individuals, the expression of genes encoding proteins related to autophagy and the antimicrobial activity of platelets under specific conditions was extremely elevated.

**Figure 5:**
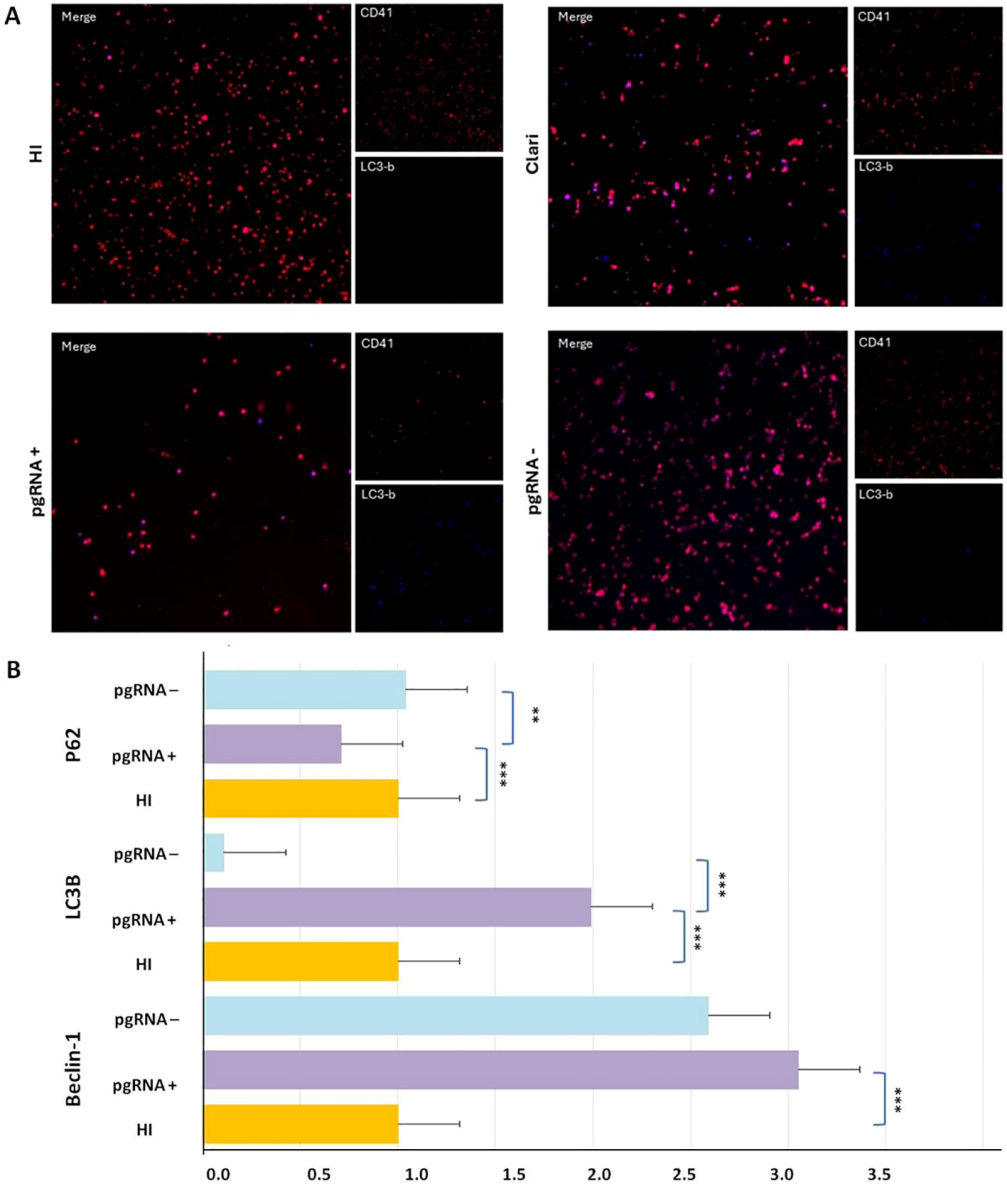
**(A)** Immunofluorescence of activated platelets with HBV pgRNA depicting the presence of LC3-B and the platelet surface index CD41 under four different conditions: stimulation with (I) healthy serum (negative control), (II) clarithromycin (positive control), (III) CHB HBV pgRNA-positive serum, and (IV) CHB pgRNA-negative serum. LC3-B is colored blue, and the CD41 platelet surface index is colored red. **(B)** Bar plot depicting the fold change in autophagy through the BECLIN-1, LC3-B, and P62 proteins in platelets activated with HBV pgRNA. For statistical significance and evaluation of the results, the cohort was repeated for 12 distinct samples. ** equal to 0.01; *** equal to <0.01.

Platelet activation is attributed to chronic inflammation, which occurs in the liver microenvironment. This activation is related to the autophagy pathway; therefore, we studied the main autophagy genes after activation with HBV pgRNA+/- serum in healthy platelets. Some of them included BECLIN-1, LC3-B, and P62. These genes were found to be differentially expressed in platelets after incubation with CHB patient serum. The HBV pgRNA-positive sera used to stimulate the platelets were significantly associated with autophagy markers, such as BECLIN-1 and LC3-B. More precisely, these genes were significantly elevated (*p* < 0.001). In particular, the level of LC3-B was almost twofold greater in HBV pgRNA-positive CHB patients than in HBV pgRNA-negative CHB patients (**Figures 5A, B**). In contrast, P62 expression was downregulated in the HBV pgRNA-positive samples compared with the HBV pgRNA-negative samples from the CHB patients (*p* = 0.001). This expression motif depicts the existence of autophagy in platelets after specific serum pgRNA-positive conditions are established. Autophagy usually occurs through the overexpression of membrane-located LC3-B and the downregulation of the P62 nuclear protein during autophagolysosome construction (19). The existence of autophagy in activated platelets is justified because of the unconditional presence of autophagy at baseline levels, which are elevated after their exposure to stimuli. Finally, the presence of platelets in the chronic HBV microenvironment could be one of the possible causes of immunothrombosis (34).

### 3.4 Platelet antimicrobial and migratory action

**Figure 6:**
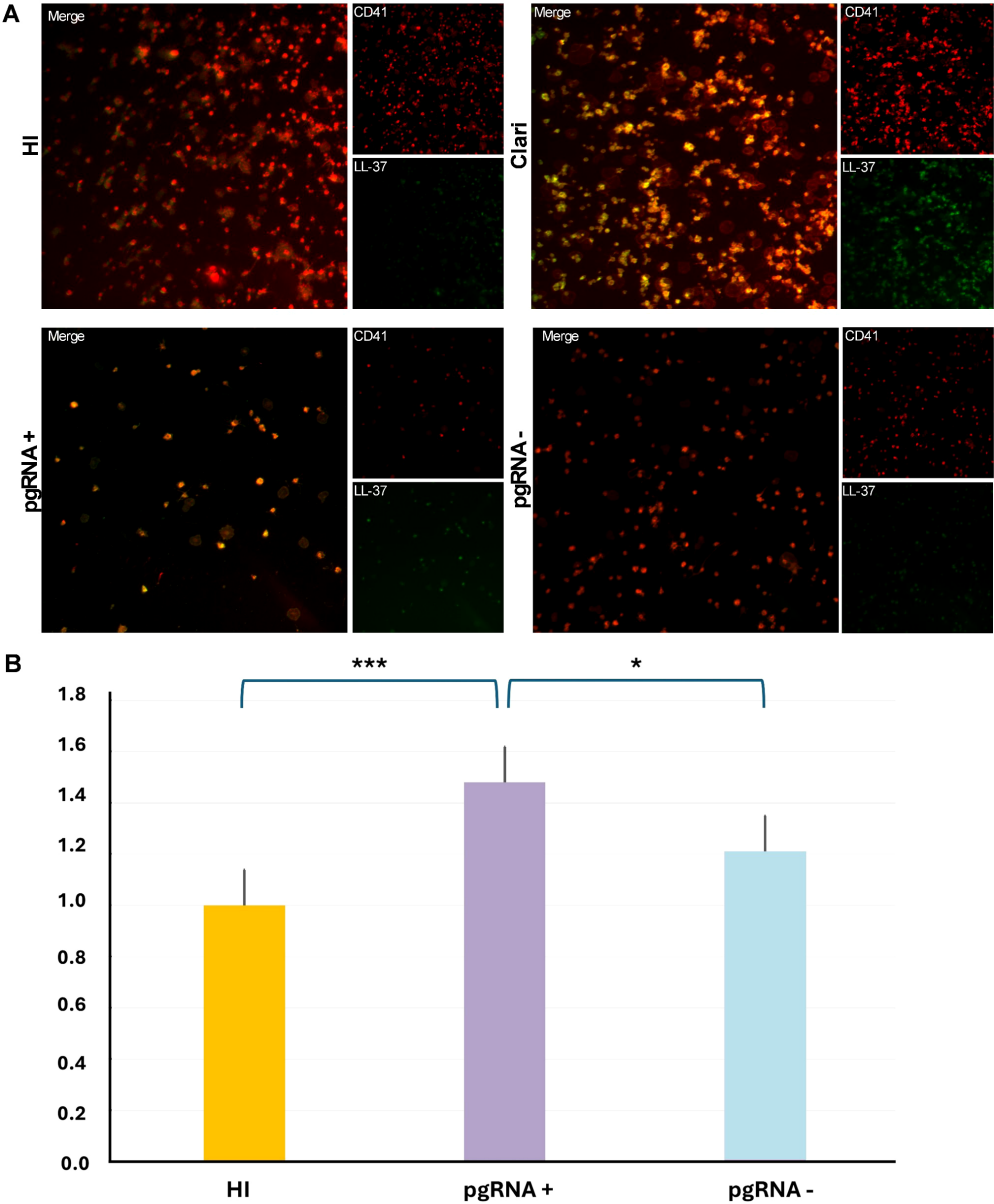
**(A)** Immunofluorescence of LL-37 in activated platelets stimulated with (I) healthy serum, (II) clarithromycin, (III) HBV pgRNA-positive serum, and (IV) HBV pgRNA-negative serum. LL-37 is depicted in green, and the CD41 surface platelet index is depicted in red. **(B)** LL-37-fold change in activated platelets treated with (I) healthy serum, (II) HBV pgRNA-positive serum, and (III) HBV pgRNA-negative serum. For statistical significance and evaluation of the results, the cohort was repeated for 12 distinct samples. * equal to 0.05; *** equal to < 0.01.

Moreover, activated platelets have a specific antimicrobial action that is derived from the released granules. These a-granules usually contain antimicrobial peptides, such as LL-37. LL-37 was overrepresented in activated and HBV pgRNA-positive serum platelets (*p* = 0.01; **Figures 6A, B**). LL-37 antimicrobial activity is present in activated platelets and seems to be released in the liver microenvironment. However, the effect of LL-37 could be reciprocal because according to literature the LL-37 has been proven to enhance both the antimicrobial function of platelets and the development of a fibrotic phenotype in liver.

However, our experiments revealed neither antimicrobial action of the activated platelets nor migration of fibroblasts after treatment with the activated platelets’ supernatant. For example, *E. coli* cultures treated with the supernatant of stimulated platelets showed an absence of inhibition of *E. coli* growth after overnight incubation (**Supplementary Figure 2A**). In addition, the migration of wounded fibroblasts was not observed in either those treated with HBV pgRNA-positive platelet supernatant or those treated with HBV pgRNA-negative supernatants (**Supplementary Figure 2B**).

### 3.5 Platelet and neutrophil interactions

The immunothrombosis profile depicted from the activation of platelets in the presence of the HBV pgRNA displays the status of the fibrotic profile in the newly entered NA-treated CHB patients. However, there are no direct stimuli from activated platelets to liver mesenchymal cells. As a result, we studied the contribution of platelet and neutrophil interactions to the liver microenvironment.

**Figure 7:**
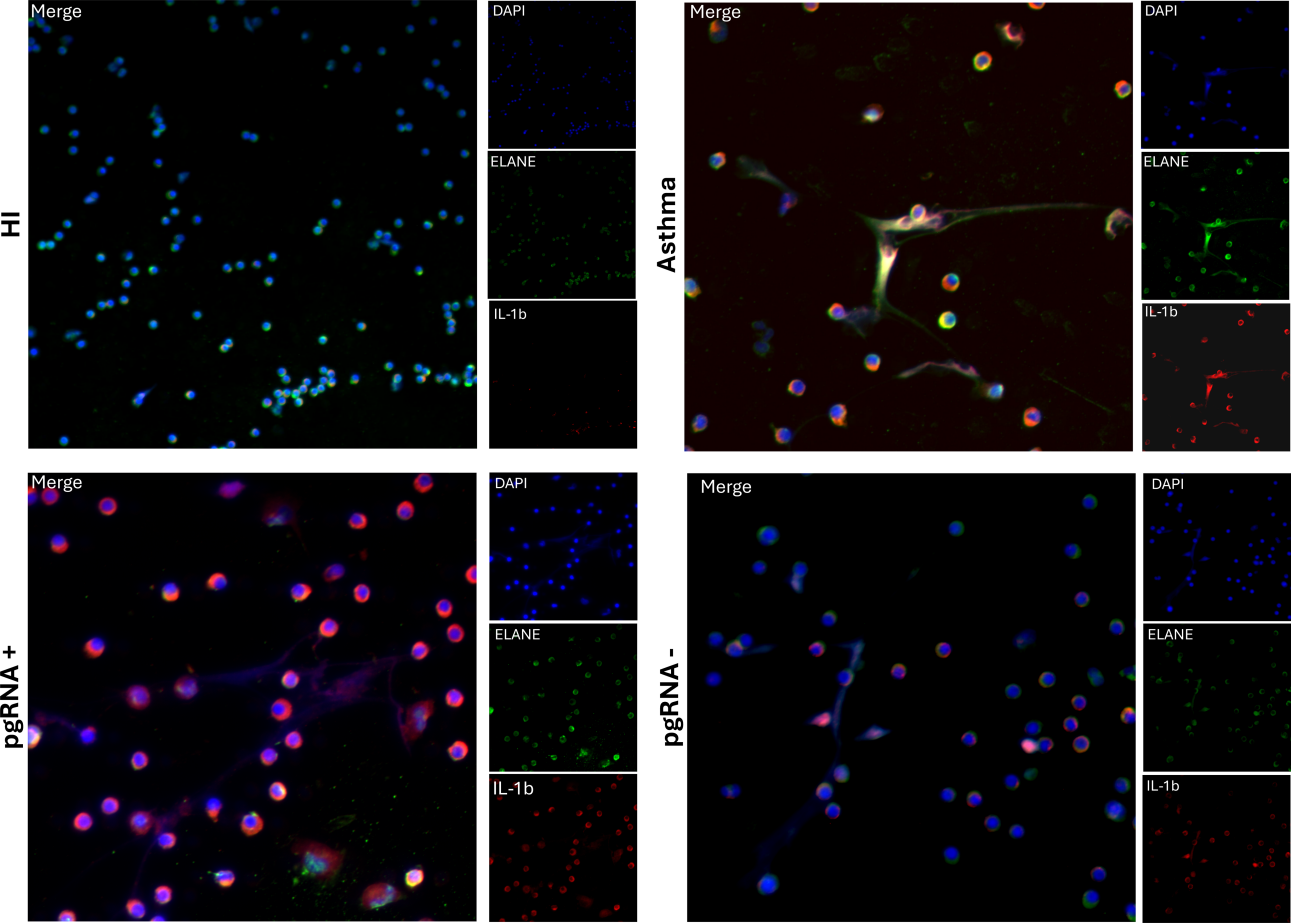
Immunofluorescence of neutrophils treated with (I) healthy serum, (II) asthma serum, (III) supernatant from platelets stimulated with HBV pgRNA-positive serum, and (IV) supernatant from platelets stimulated with HBV pgRNA-negative serum. IL-1β is colored red, neutrophil elastase (ELANE) is colored green, and DAPI is colored blue.

**Figure 8:**
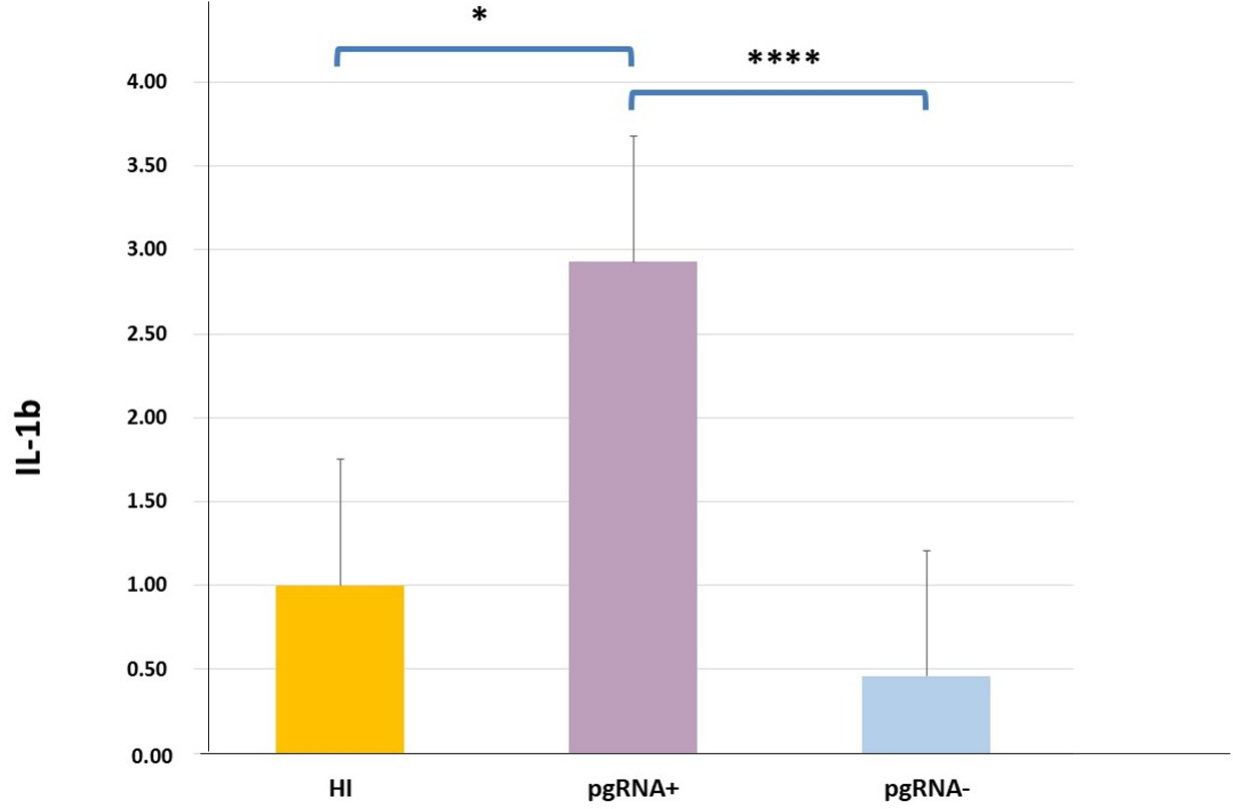
IL-1β fold change in neutrophils treated with (I) healthy serum, (II) supernatant from platelets stimulated with HBV pgRNA-positive serum, and (III) supernatant from platelets stimulated with HBV pgRNA-negative serum. For statistical significance and evaluation of the results, the cohort was repeated for 12 distinct samples. * equal to 0.05; **** equal to <0.001.

The coculture of neutrophils with the supernatant of HBV pgRNA-activated platelets revealed a proinflammatory phenotype that coexisted in the liver microenvironment through the expression of IL-1β. As shown in **Figure 7**, the activated neutrophils did not release IL-1β or form NET structures; they were instead filled with IL-1β. However, the neutrophils treated with the supernatant of platelets in the absence of the HBV pgRNA index did not present any distinct signs of IL-1β. In addition, the activated neutrophils do not present any netotic profile; they remain intact instead compared with the asthma conditions (**Figure 7**). To better validate the abovementioned results, the IL-1β levels were measured using quantitative real-time reverse transcription polymerase chain reaction (qRT-PCR), which revealed significant increases in the levels of IL-1β in neutrophils stimulated with platelet supernatant first treated with HBV pgRNA-positive serum but not in those treated with HBV pgRNA-negative serum (**Figure 8**).

**Figure 9:**
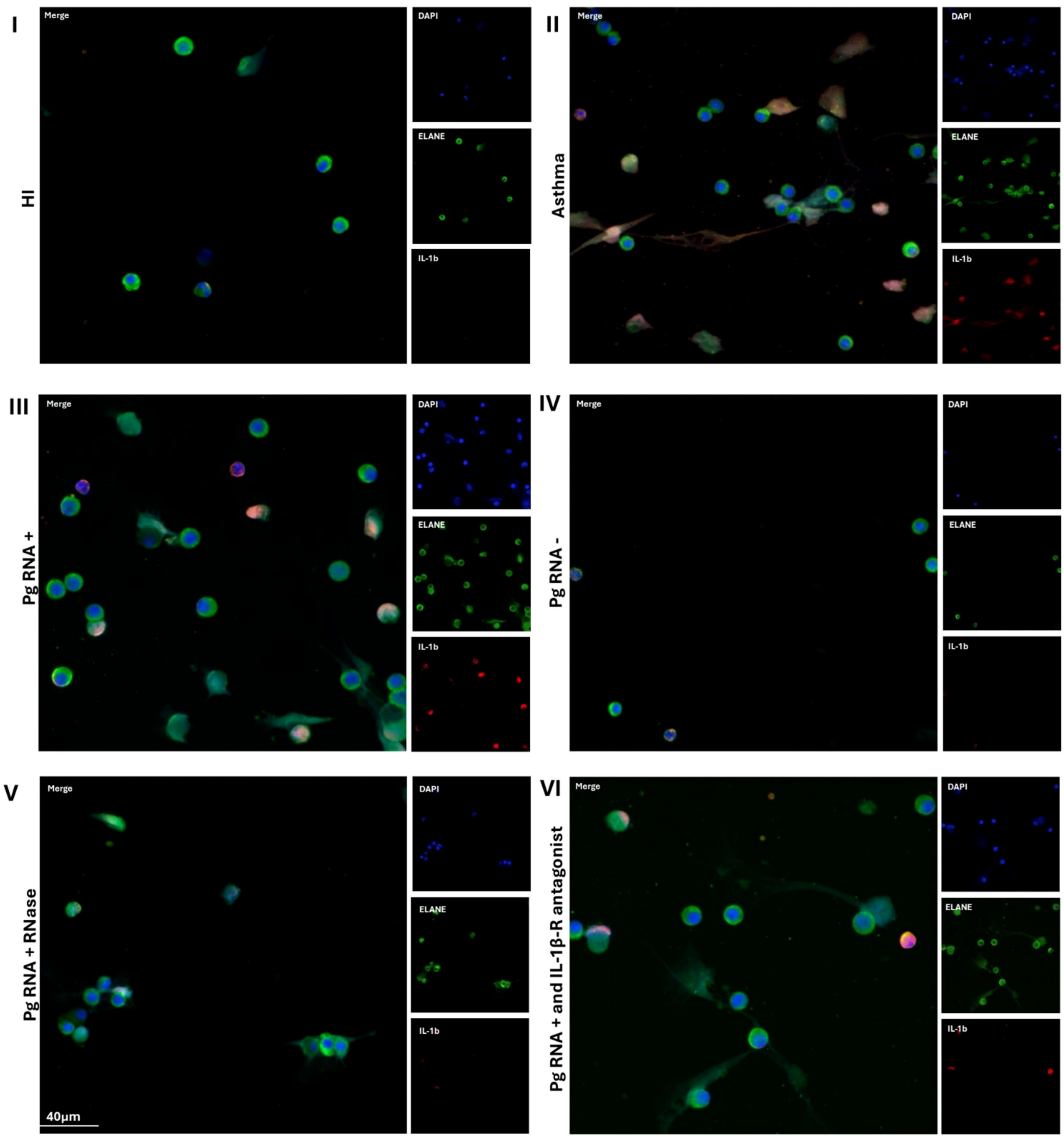
Immunofluorescence of neutrophils treated with (I) healthy serum, (II) asthma serum, (III) supernatant from platelets stimulated with HBV pgRNA-positive serum, (IV) supernatant from platelets stimulated with HBV pgRNA-negative serum, (V) supernatant from platelets stimulated with HBV pgRNA-positive serum and RNase, and (VI) HBV pgRNA-positive serum and IL-1β-receptor antagonist (anakinra). IL-1β is colored red, neutrophil elastase (ELANE) is colored green, and DAPI is colored blue.

Although neutrophils are not subjected to a netotic phenotype, there is a significant expression of proinflammatory cytokines that could conribute to chronic persistent sterile inflammation in the liver microenvironment. To further assess whether this phenotype derives from the HBV pgRNA-positive serum, we incubated healthy platelets with RNA-depleted serum, and their supernatant was given to healthy neutrophils. As expected, the presence of IL-1β was eliminated in the neutrophils’ cytoplasm (**Figure 9**—V). Possible circulating IL-1β interference, which may lead to the IL-1β-induced phenotype inside the neutrophils, suggested the addition of a IL-1β receptor antagonist in the HBV pgRNA-positive serum before the pretreatment of the healthy platelets. The readout of this incubation showed that neutrophils incubated with the platelets’ supernatant had increased levels of IL-β. This finding suggests the ability of neutrophils to express and not to acquire circulated IL1-β from their environment when induced with the supernatant derived from HBV pgRNA-positive-stimulated platelets (**Figure 9**—VI).

**Figure 10:**
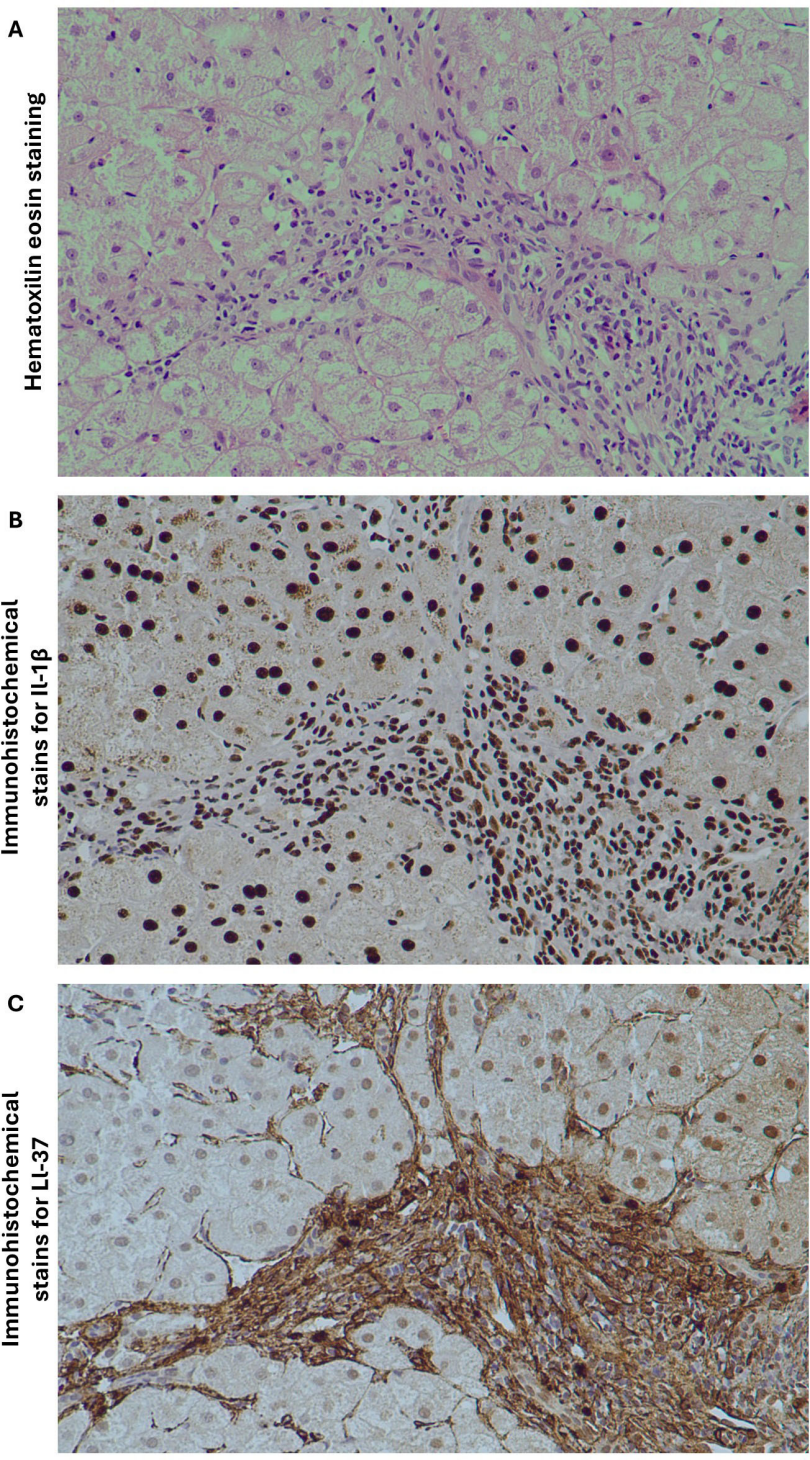
Immunohistochemical staining of liver tissue obtained from patients with CHB: **(A)** hematoxylin and eosin staining, **(B)** IL-1β staining, and **(C)** LL-37 staining.

Moreover, we later stained a liver tissue obtained from a CHB patient to confirm the inflammation profile that we had observed through the *in vitro* experiments. To our surprise, the tissue is infiltrated by neutrophils, as observed by hematoxylin and eosin staining. There are abundant IL-1β and LL-37 in the liver microenvironment showing a particular localization inside the neutrophils (**Figure 10**). Those findings are in line with previous *in vitro* observations and confirm the presence of a sterile inflammation in the liver microenvironment as well.

**Figure 11:**
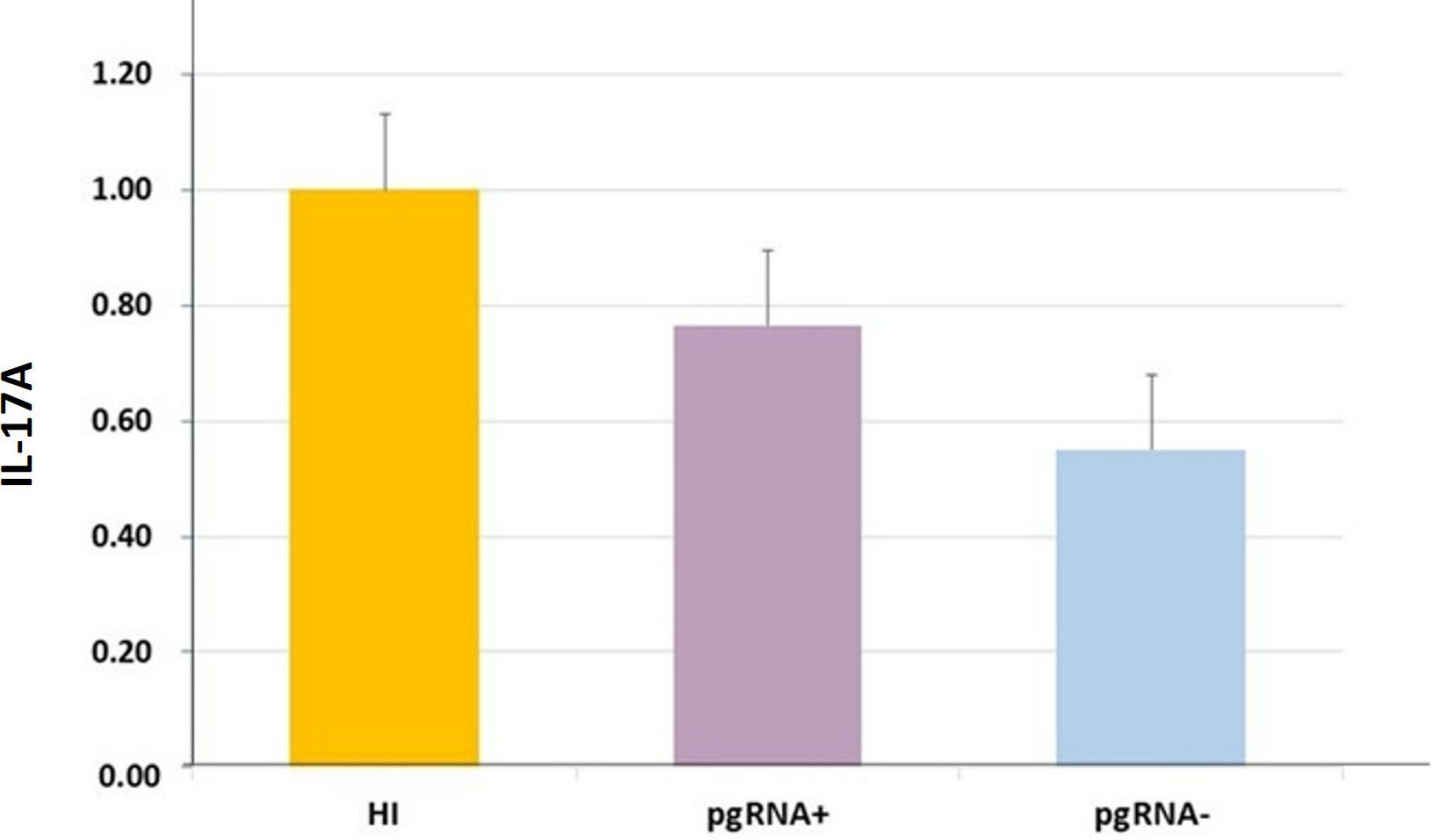
IL-17A fold change in neutrophils treated with (I) healthy serum, (II) supernatant from platelets stimulated with HBV pgRNA-positive serum, or (III) supernatant from platelets stimulated with HBV pgRNA-negative serum. For statistical significance and evaluation of the results, the cohort was repeated for 12 distinct samples.

Finally, we tested the fibrotic profile of activated neutrophils by examining the level of IL-17A. The fibrotic phenotype is absent from neutrophils, which is the first case of elevated IL-1β levels, as there is a decrease in IL-17A (**Figure 11**). This combinational expression motif of cytokines provides information about chronic illness status and describes a dynamic condition that can be easily triggered and can evolve into illness exacerbation.

## 4 Discussion

In this study, CHB patients with chronic HBeAg-negative hepatitis receiving NA treatment were investigated (ENEG). We examined all of our patients for biological and viral HBV indices and calculated the fibrotic score FIB-4 and the HCC predictive score PAGE-B. The HBV pgRNA was apparently positively related to the ALT, AST, HBV DNA, and HBsAg levels and negatively related to the therapy duration. Given the prognosis and the evaluation, which is added by the HBV pgRNA in the FIB-4 and PAGE-B score, no difference was demonstrated. Moreover, platelet enumeration had no significant variance between the HBV pgRNA-positive and HBV pgRNA-negative populations (**Supplementary Table 7**) (35).

After the clinical data demonstration, we analyzed the transcriptome profile of our patients. The two transcriptomic analyses revealed common pathways, which were further explored through *in vitro* experiments. The pathways described mostly involve procedures such as (I) platelet activation and aggregation, (II) the regulation of the cellular response to oxidative stress, and (III) neutrophil degranulation, which contribute to chronic illness. This study provides new insights into disease progression and suggests novel pathways related to CHB staging. Most studies describe HBV illness staging through single-cell RNA-seq and involve mostly cells and functions of adaptive immunity. Studies describing the broad transcriptomic profile of CHB patients, including the use of circulating agents, are limited (31). Our transcriptomic data were in line with previously reported data by Karaoglu et al. (36).

In the study by Karaoglu et al., the presence of common pathways detected between the CHB liver biopsies and our CHB plasma samples is depicted. Those pathways, such as “neutrophil migration”, “response to oxidative stress”, “RNA metabolism”, and “response to virus”, reveal a distinguished contribution to chronic illness (32). Nevertheless, there are significant differences between the two studies, as our results discuss HBV pgRNA interactions in CHB patients and relate them to a supplementary analysis of HBV-related HCC pathways. The differentially expressed genes that caught our attention subsequently contributed to “platelet activation signaling and aggregation” and “neutrophil degranulation”.

Innate immunity mechanisms are significantly suppressed by HBV during infection, resulting in insufficient IFNa/b production and the downregulation of TLRs (33, 34). This is a primary mechanism of partial stealth during HBV infection. During this process, a variety of cell populations interact with each other to defend against HBV. The role of platelets in inflammation is relevant to the procedures of immunothrombosis and hemostatic impairment. According to Enger et al., chronic hepatitis B is a possible thrombotic risk factor, as there is a high incidence of arterial or venous thromboembolic events in patients suffering from cirrhosis (37).

Nevertheless, the role of activated platelets in CHB could be further studied. STEMI is a pathological condition in which activated platelets promote functional pathways through interactions with polymorphonuclear neutrophils (PMNs). Stakos et al. reported that activated platelets from the infarct-related coronary artery (IRA) were able to induce NET release in healthy PMNs, whereas isolated platelets from non-IRA patients did not exhibit NET formation (36). The abovementioned experimental and clinical assumptions contributed to our main study regarding platelet activation. For the first time, we suggest that serum derived from HBV pgRNA-positive CHB patients activated platelets in an autophagy-dependent manner. *In vitro* experimental workflows showed significantly elevated LC3-B and BECLIN-1 levels in contrast to the P62 nuclear protein. These results indicate that the capacity of HBV pgRNA could efficiently trigger platelet activation during the early Ishak 0/1 stages rather than the advanced stages, as transcriptionally healthy hepatocytes contribute to the resources of HBV pgRNA (**Supplementary Table 7**) (35). However, more high-throughput studies should be conducted to assess the capacity of HBV pgRNA in chronic diseases. Since platelets activated with HBV pgRNA-positive serum have been shown to activate the autophagy machinery, we investigated the protein components in platelets. We detected an increase in LL-37 levels in platelets. To assess the *de novo* ability of platelets to synthesize LL-37 instead of receiving it from their environment, we analyzed LL-37 mRNA levels in activated platelets. Pircher et al. reported that LL-37 primes platelets to mediate arterial thrombosis and tissue inflammation (15), whereas the ability to synthesize platelets *de novo* was reported for the first time in this article. Our group previously reported that LL-37’s antimicrobial and antibiofilm effects play crucial roles in tissue remodeling and fibrosis (38). Given that platelet-mediated LL-37 production and its functionality have not yet been reported, we further investigated the functionality of platelet-produced LL-37. Surprisingly, the increased LL-37 levels did not demonstrate any functional outcome of the LL-37 peptide, both in terms of its antimicrobial activity and its fibrotic activity (**Supplementary Figure 2**). This finding comes in agreement with the chronic inflammation in T2D patients according to Arampatzioglou et al. Even if well-controled chronic T2D patients can spontaneously release NETs and produce LL-37, the functionality of the latter lacks both its antimicrobial and its fibrotic effect. However, the induction of LL-37 production with clarithromicyn reprogrammed both the antimicrobial and the fibrotic phenotype in well-controlled T2D rather than in naïve T2D (39).

A previous study reported that neutrophils, especially those secreting IL-1β, are implicated in HBV pathophysiology, both in early and late disease stages, and mediate inflammatory and/or fibrotic events (38). This finding is in line with the enriched presence of IL-1β in the infiltrated neutrophils present in the CHB liver tissue. The role of the interaction between platelets and neutrophil/IL-1β-bearing NETs has yet to be elucidated. Since HBV pgRNA-positive serum activates platelets, we further investigated the ability of platelets to interact with neutrophils. We found that neutrophils treated with platelet-derived supernatant, which was first stimulated with HBV pgRNA-positive serum, could concomitantly induce a proinflammatory phenotype in neutrophils bearing IL-1β but not secreting IL-1β because of the absence of NET formation. In addition, we used IL-1β-bearing neutrophils to study the expression of the fibrotic phenotype through IL-17A. IL-17A has already been identified as a liver fibrogenesis activator (40). However, we did not report any overexpression of IL-17A in our samples. Consequently, in CHB patients, the presence of fibrotic events has not been described in HBV pgRNA-positive patients, validating the transcriptional activity of healthy hepatocytes.

Moreover, we sought to demonstrate whether the IL-1β induction in neutrophils is completely HBV pgRNA-positive dependent by both excluding the interference of serum contained IL-1β and depleting the HBV pgRNA in the serum. The treatment with anakinra and RNase of the HBV pgRNA-positive serum could strongly support our view that the HBV pgRNA has a concominant role in the liver’s sustained inflammation. The platelets’ supernatant derived after treatment with the HBV-pgRNA-depleted serum could not trigger the expression of IL-1β in the healthy neutrophils. However, this theory is not validated by the induction of LL-37 in the platelets that follow the autophagy-dependent activation. The HBV pgRNA could be the inducer for the platelets’ activation, but there are more parameters on how the activated platelets interact with the neutrophils in the liver microenvironment and these should be further explored.

To summarize, there are limitations to this study. Initially, we performed transcriptome analysis in a small group of patients. Consequently, we were unable to perform analyses with different disease phenotypes. Furthermore, the role of IL-1β in CHB could be further studied for its relation with the autophagy-dependent pathway in platelets and whether there is a stimulator between the platelet–neutrophil interaction that could trigger inflammation in the liver microenvironment. Finally, the limited availability of biopsies did not allow us to verify these results on the side of infection.

**Figure 12:**
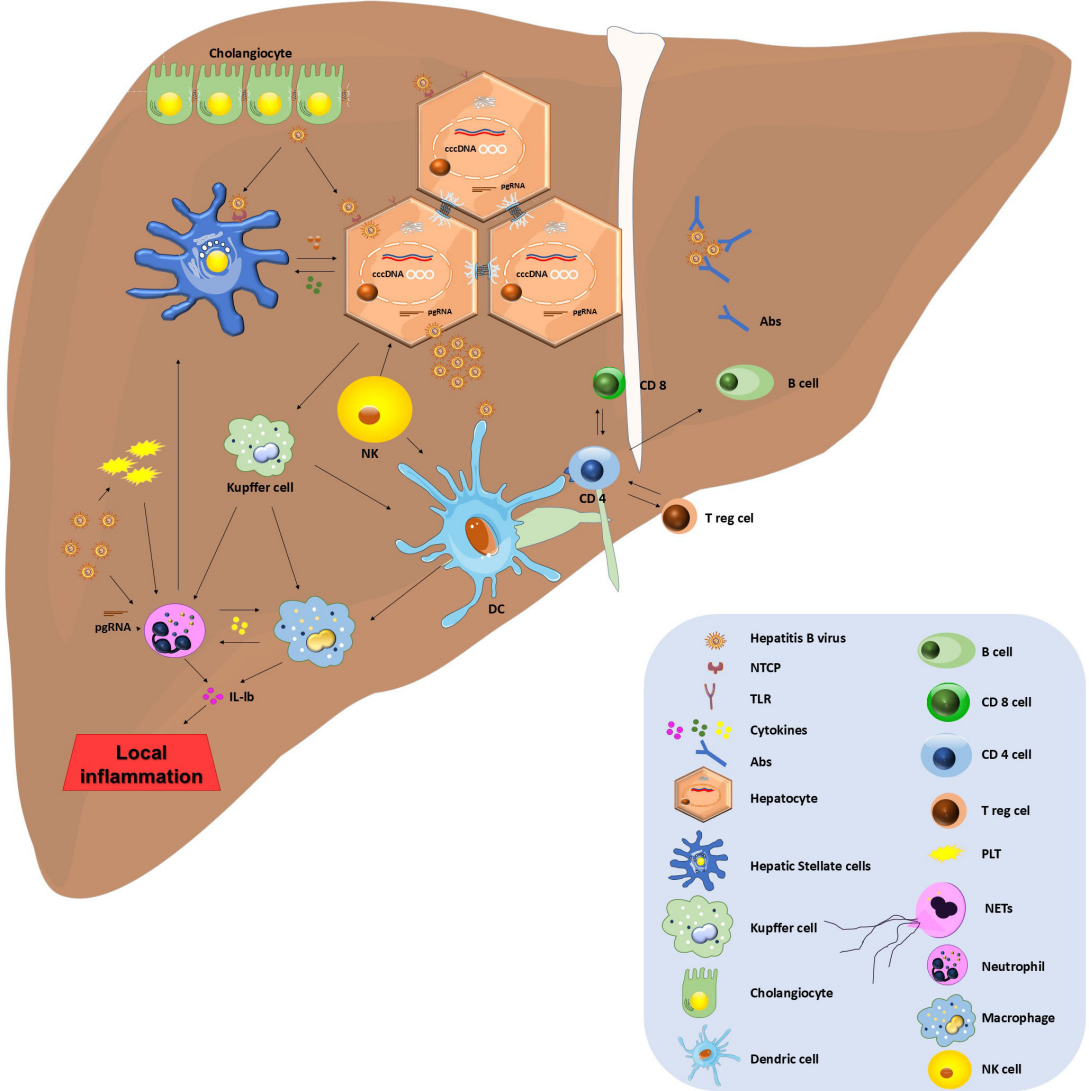
Depicting innate immunity in liver inflammation, in cirrhosis, in HCC, and in chronic hepatitis **(B)** The continuous presence of innate immunity to the virus activates immunological mechanisms in the liver by inviting a variety of immunological cells and agents in the liver microenvironment. The activation of proinflammatory regulators, such as IL-1β, conserves local inflammation by stimulating stellate cells. In addition, the continuous enhancement of this proinflammatory stimulus by the presence of cccDNA and pgRNA triggers illness development into fibrosis, cirrhosis, and HCC. KCs, Kupffer cells; HSCs, hepatic stellate cells; DCs, dendritic cells; NKs, natural killer; cccDNA, covalently closed circular DNA; pgRNA, pregenomic RNA; HCC, hepatocellular carcinoma.

In conclusion, by summarizing the data presented in this article, we propose the pathophysiological mechanisms of CHB immunity depicted in **Figure 12**.

## Data Availability

The dataset (GSE316961) used for this study can be found in the Gene Expression Omnibus (https://www.ncbi.nlm.nih.gov/geo/query/acc.cgi?acc=GSE316961).
