# Supplementary material for: HBV pgRNA induces chronic inflammation in an IL-1β-dependent manner
Source: Front Immunol. 2026 Jun 22;17:1812831. doi: 10.3389/fimmu.2026.1812831 (PMC13333505; doi:10.3389/fimmu.2026.1812831)
Supplement: Supplementary file 1 [file DataSheet1.docx]

Table 1 Thermal profile of RT-PCR for the genes LC3-B, P62 and LL-37

| *Program* | *Temperature* | *Time* | *Cycles* |
| --- | --- | --- | --- |
| *Denaturation* | *95oC* | *10’* | *1* |
| *Amplification* | *95 oC* | *5’’* | *50* |
|  | *62 oC* | *5’’* |  |
|  | *72 oC* | *15’’* |  |
| *Melting* | *95 oC* | *20’’* | *1* |
|  | *40 oC* | *20’’* |  |
|  | *85 oC* | *∞* |  |
| *Cooling* | *40 oC* | *30’’* | *1* |

*Table 2 Thermal profile of RT-PCR for the genes GAPDH,and BECLIN-1*

| *Program* | *Temperature* | *Time* | *Cycles* |
| --- | --- | --- | --- |
| *Denaturation* | *95oC* | *10’* | *1* |
| *Amplification* | *95 oC* | *5’’* | *50* |
|  | *56 oC* | *5’’* |  |
|  | *72 oC* | *15’’* |  |
| *Melting* | *95 oC* | *20’’* | *1* |
|  | *40 oC* | *20’’* |  |
|  | *85 oC* | *∞* |  |
| *Cooling* | *40 oC* | *30’’* | *1* |

*Table 3 Thermal profile of RT-PCR for the genes IL-1b*

| *Program* | *Temperature* | *Time* | *Cycles* |
| --- | --- | --- | --- |
| *Denaturation* | *95oC* | *10’* | *1* |
| *Amplification* | *95 oC* | *5’’* | *50* |
|  | *58 oC* | *5’’* |  |
|  | *72 oC* | *15’’* |  |
| *Melting* | *95 oC* | *20’’* | *1* |
|  | *40 oC* | *20’’* |  |
|  | *85 oC* | *∞* |  |
| *Cooling* | *40 oC* | *30’’* | *1* |

*Table 4 Thermal profile of RT-PCR for the genes IL-17A*

| *Program* | *Temperature* | *Time* | *Cycles* |
| --- | --- | --- | --- |
| *Denaturation* | *95oC* | *10’* | *1* |
| *Amplification* | *95 oC* | *5’’* | *50* |
|  | *53 oC* | *5’’* |  |
|  | *72 oC* | *15’’* |  |
| *Melting* | *95 oC* | *20’’* | *1* |
|  | *40 oC* | *20’’* |  |
|  | *85 oC* | *∞* |  |
| *Cooling* | *40 oC* | *30’’* | *1* |

*
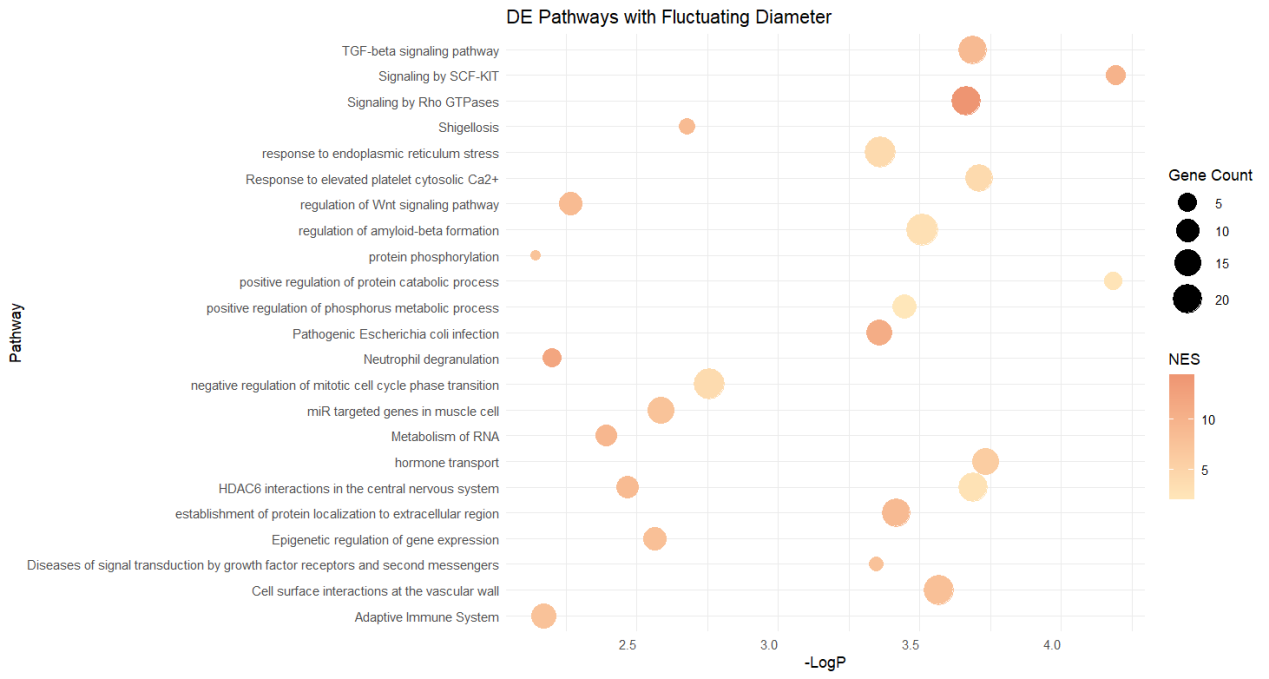
*

Figure 1 Bubble plot of enhanced pathways derived from the differentially expressed genes (DE), for the HBV pgRNA+/- GSEA.


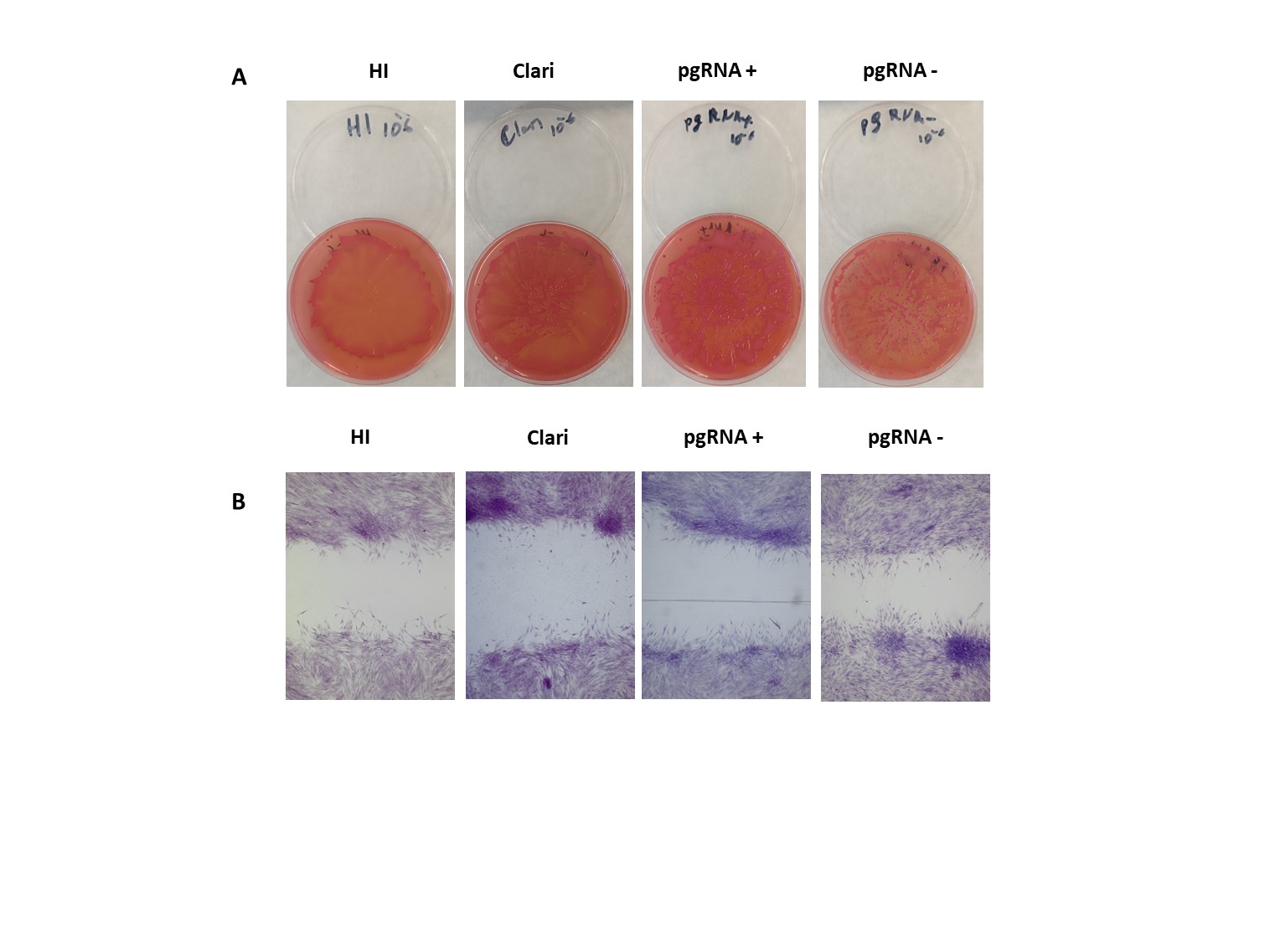


Figure 2 A. Escherichia coli cultures after 24h incubation with the supernatant (SN) of activated platelets with; i) SN HI PLTs, ii) SN Clari PLTs; stimulation with clarithromycin, iii) SN HBV pgRNA+ PLTs; stimulation with positive HBV pgRNA serum, iv) SN HBV pgRNA- PLTs; stimulation with negative HBV pgRNA serum,

B. Fibroblasts’ migration assay after stimulation with SN from activated platelets with i) SN HI PLTs, ii) SN Clari PLTs; stimulation with clarithromycin, iii) SN HBV pgRNA+ PLTs; stimulation with positive HBV pgRNA serum, iv) SN HBV pgRNA- PLTs; stimulation with negative HBV pgRNA serum

Table 5 Metrics of RNA quality and quantity.

| Sample | Biological sample | Nanodrop | A260/280 | A260/230 | Qbit | Comments |
| --- | --- | --- | --- | --- | --- | --- |
| IND44 M | PLASMA | 282.2 ng/ul | 2.04 | 2.2 | 250.6 ng/ul | Healthy |
| IND47 M | PLASMA | 164.04 ng/ul | 1.9 | 2.01 | 125.8 ng/ul | Healthy |
| IND27 F | PLASMA | 156.69 ng/ul | 1.89 | 1.73 | 126.7 ng/ul | Healthy |
| IND61 F | PLASMA | 178.14 ng/ul | 1.84 | 1.94 | 159.4 ng/ul | Healthy |
| MOAP | PLASMA | 169.11 ng/ul | 1.85 | 2.39 | 147.2 ng/ul | Negative pgRNA |
| OMEL | PLASMA | 180.55 ng/ul | 1.97 | 1.83 | 163.2 ng/ul | Negative pgRNA |
| DODI | PLASMA | 160.7 ng/ul | 2.11 | 1.17 | 136.4 ng/ul | Negative pgRNA |
| TZELEP BACK | PLASMA | 151.17 ng/ul | 1.97 | 1.49 | 121.3 ng/ul | Negative pgRNA |
| MPLO BACK | PLASMA | 161.1 ng/ul | 1.99 | 1.87 | 136.2 ng/ul | Positive pgRNA |
| KEAMSE | PLASMA | 166.57 ng/ul | 1.93 | 1.85 | 124.9 ng/ul | Positive pgRNA |
| MIEG | PLASMA | 144.4 ng/ul | 1.82 | 1.67 | 132.2 ng/ul | Positive pgRNA |
| XANA | PLASMA | 145.10 ng/ul | 1.89 | 2.21 | 120.7 ng/ul | Positive pgRNA |

Table 6 Clinical variables related to HBV pgRNA prevalence in chronic Hepatitis B patients, negative for the HBeAg.

| **Variables** | **Overall (n=88)** | **HBV pgRNA**  **negative (n=72)** | **HBV pgRNA positive (n=16)** | **p-value** |
| --- | --- | --- | --- | --- |
| **Age** |  |  |  |  |
| Years (mean ± SD) | 58.6 ± 12.4 | 60.8 ± 11.4 | 49.0 ± 12.2 | <0.001 |
| **Gender** |  |  |  |  |
| Male | 43 (49) | 35 (49) | 8 (50) | 0.920 |
| Female | 45 (51) | 37 (51) | 8 (50) |  |
| **AST** |  |  |  |  |
| U/L; median (IQR) | 24 (19 – 31) | 22 (18 – 27) | 33 (25 – 39) | 0.001† |
| **(AST)^1/2^** |  |  |  |  |
| median (IQR) | 4.8 (4.4 – 5.5) | 4.7 (4.2 – 5.2) | 5.7 (5.0 – 6.2) | 0.001† |
| **ALT** |  |  |  |  |
| U/L; median (IQR) | 22(17 – 34) | 22 (16 – 31) | 36 (25 – 55) | 0.001† |
| **(1/ALT)^1/2^** |  |  |  |  |
| (L)^1/2^; median (IQR) | 0.21 (0.17 – 0.24) | 0.22 (0.18 – 0.25) | 0.17 (0.14 – 0.20) | 0.001† |
| **AST/ALT** |  |  |  |  |
| median (IQR) | 1.0 (0.8 – 1.3) | 1.0 (0.8 – 1.3) | 0.9 (0.9 – 1.7) | 0.854† |
| **(AST/ALT)^1/2^** |  |  |  |  |
| median (IQR) | 1.0 (0.9 – 1.1) | 1.0 (0.9 – 1.1) | 1.0 (0.9 – 1.3) | 0.854† |
| **PLT** |  |  |  |  |
| 10^9^/L; median (IQR) | 222 (162 – 239) | 204 (164 – 236) | 231 (132 – 247) | 0.665† |
| **1/PLT** |  |  |  |  |
| μL; median (IQR) | 4.45 (3.89 – 5.64) | 4.53 (3.96 – 5.65) | 4.12 (3.46 – 5.14) | 0.215† |
| **HBsAg** |  |  |  |  |
| median (IQR) | 522 (56 – 3003) | 309 (39 – 2337) | 3003 (319 – 5446) | 0.002† |
| **HBV viral load** |  |  |  |  |
| copies/ml; median (IQR) | 0 (0 – 21) | 0 (0 – 0) | 783 (0 – 2968) | <0.001† |
| **HBV viral load presence** |  |  |  |  |
| No | 66 (75) | 61 (85) | 5 (31) | <0.001‡ |
| Yes | 22 (25) | 11 (15) | 11 (69) |  |
| **HBV pgRNA** |  |  |  |  |
| **copies/ml; median (IQR)** | 1.4 (1.0 – 1.9) | 0 (0 – 0) | 776 (564 – 2018) | <0.001† |
| **Regimen** |  |  |  |  |
| ETV | 50 (57) | 39 (54) | 11 (69) | 0.287 |
| TDF | 38 (43) | 33 (46) | 5 (31) |  |
| **Duration of therapy** |  |  |  |  |
| months; median (IQR) | 114 (39 – 168) | 144 (60 – 168) | 18 (5 – 72) | <0.001† |
| **Therapy > 6 years** |  |  |  |  |
| No | 37 (42) | 24 (33) | 13 (81) | 0.001‡ |
| Yes | 51 (58) | 48 (67) | 3 (19) |  |
| **FIB-4 score** |  |  |  |  |
| median (IQR) | 1.3 (1.0 – 1.9) | 1.3 (1.0 – 1.9) | 1.1 (0.9 – 2.2) | 0.566† |
| **Advanced fibrosis (METAVIR stage F3/4) likely**  **(FIB-4 score ≥2.67)** |  |  |  |  |
| No | 79 (90) | 66 (92) | 13 (81) | 0.355‡ |
| Yes | 9 (10) | 6 (8) | 3 (19) |  |
| **Approximate Ishak**  **fibrosis stage** |  |  |  |  |
| 0-1 | 53 (60) | 43 (60) | 10 (62) | 0.066‡ |
| 2-3 | 29 (33) | 26 (36) | 3 (19) |  |
| 4-6 | 6 (7) | 3 (4) | 3 (19) |  |
| **Cirrhosis**  **(Fibroscan documented)** |  |  |  |  |
| No/missing | 75 (85) | 62 (86) | 13 (81) | 0.698‡ |
| Yes | 13 (15) | 10 (14) | 3 (19) |  |
| **PAGE-B** |  |  |  |  |
| median (IQR) | 12 (8 – 16) | 12 (10 – 16) | 9 (5 – 14) | 0.459† |
| **PAGE-B stage** |  |  |  |  |
| 1 (≤9) | 26 (30) | 21 (29) | 5 (31) | 0.979 |
| 2 (10 - 17) | 46 (52) | 38 (53) | 8 (50) |  |
| 3 (≥18) | 16 (18) | 13 (18) | 3 (19) |  |

† Independent-Samples Mann-Whitney U test

‡ Fisher’s exact test

AST; Aminotransferase

ALT; Alanine transaminase

PLT; Platelets

Table 7 Ishak score in chronic hepatitis B, HBeAg negative patients in accordance to PLTs and Age

| **Variables** | **Ishak 0-1** | **Ishak 2-3, 4/5/6** | **p-value** |
| --- | --- | --- | --- |
| **PLTs 10^9^/L** | 251.6 (159-392) | 174.8 (87-265) | p <0.0001† |
| **Age (years)** | 54.78 (27-74) | 66.19 (40-82) | p <0.0001† |
| **HBV pgRNA (copies/ml)** | 303 (0.00-2725) | 134.8 (0.00-2280) | p= 0.027 |

† Independent-Samples Mann-Whitney U test
